# Clinical Outcomes in Hormone Replacement Therapy (HRT)-Frozen Embryo Transfer (FET) Protocol Increased by Administering Gonadotropin-Releasing Hormone Agonist (GnRH) in the Initial Stage of the Luteal Phase: A Retrospective Analysis

**DOI:** 10.7759/cureus.53877

**Published:** 2024-02-08

**Authors:** Aamir Mahmood, Li Tan

**Affiliations:** 1 Reproductive Medicine, The Second Affiliated Hospital of Zhengzhou University, Zhengzhou, CHN

**Keywords:** luteal phase support, stimulation assisted cycle, natural cycle, freeze-to-thaw embryo transfer, hormone replacement therapy

## Abstract

Objective: The objective of this study was to determine if gonadotropin-releasing hormone agonist (GnRH) administration supporting the luteal phase in frozen embryo transfer (FET) improves clinical outcomes

Methods and materials This is a retrospective cohort study and we analyzed 3515 cycles of FET at the Department of Reproductive Medicine in our hospital from February 2018 through December 2021. Patients were divided into the GnRH (triptorelin+progesterone and human chorionic gonadotropin (hCG)) group and the non-GnRHa (existing treatment without triptorelin) group. There were 1033 and 2485 cases in the above groups, respectively. Live birth rates (LBR) and clinical pregnancy rates (CPR) were contrasted in the two groups.

Results: We found greater CPR (58.00% versus 48.40%, P-value = 0.003) and LBR (52.70% versus 45.60%, P-value = 0.001) for HRT-FET cycles, and found no clinical significance for natural cycle FET (NC-FET) (58.20% versus 52.90%, P-value = 0.364 and 54.40% versus 47.00%, P-value=0.211), GnRH+HRT-FET (53.00% versus 53.00%, P-value=0.176 and 46.20% versus 47.30%, P-value=0.794), and stimulation-FET (59.30% versus 52.90%, P-value=.00.566 and 59.30% versus 47.10%, P-value=.00.247) in terms of CPR and LBR in the two groups. There was a 47% increase in CPR in the GnRH group, and there was a 33% increase in LBR in the same group.

Conclusion: During HRT-FET cycles, administering triptorelin three to four times in the existing luteal support can improve CPR and LBR, and administering triptorelin during the initial stage of the luteal phase can prove a new option for luteal support.

## Introduction

There are many protocols for endometrium preparation before frozen embryo transfer (FET). They are natural cycles (NCs), hormone replacement therapy (HRT) cycles, gonadotrophin-releasing hormone agonist (GnRH)-assisted HRT cycles, and stimulated-assisted cycles [1,2]. Each protocol has its advantages and limitations. FET cycles have gained significance, accounting for up to one-third of all assisted reproductive technology (ART) births in the United States [3].

Currently, there are many medications for luteal support in clinical practice, including progesterone, human chorionic gonadotropin (HCG), and estrogen [4]. Some studies have found that GnRH is used for luteal support therapy. GnRHa stimulation makes the pituitary gland increase the secretion of luteinizing hormone (LH) for luteal support [5]. While other researchers have shown that there is an expression of GnRH receptors on both sides of the placenta, normal endometrium, myometrium, ovaries, and testes [6], It is believed that GnRHa can affect the endometrium; local GnRH receptors exert a direct effect and can improve endometrial receptivity [7].

At present, GnRH supplementation in the luteal phase promotes luteal function, embryonic development potential, and embryonic development, but the mechanism of endometrial receptivity is still unclear.

To support the luteal phase in FET protocols, GnRH was observed to improve clinical outcomes during ART treatments at our reproductive center, but there was no published data to support this. Thus, this was a retrospective analysis of the FET cycles of patients who were taking treatment at our reproductive center to assess the effects of triptorelin in the luteal phase during FET cycles for clinical pregnancy rates (CPR) and live birth rates (LBR) and provide a basis for clinical application.

This article was previously posted to the ResearchSquare preprint server on August 28, 2023.

## Materials and methods

This was a retrospective study conducted in The Second Affiliated Hospital of Zhengzhou University, Zhengzhou, China, from February 2018 to December 2021. The study was approved by the Ethical Committee of The Second Affiliated Hospital of Zhengzhou University (protocol number: 2023105 dated April 24, 2023). All FET-assisted pregnancy protocols were covered including NC, HRT, GnRH+HRT, and stimulation cycle protocols of FET-assisted pregnancy.

Data were taken from the electronic patient record system of the hospital. Inclusion criteria were women aged 20-52 years, with a BMI of 15-41.6 kg/m^2^, anti-Mullerian hormone (AMH) of 0-59 ng/ml, infertility for a duration of 0.2-22 years, and their own oocytes and embryos. We excluded fresh cycle protocols for assisted pregnancy, oocyte donation cycles, donated embryos, and uterine malformations. Before FET, all patients signed the necessary informed consent forms.

Procedure

In our reproductive center, we mostly use the NC, HRT-FET, GnRH-HRT, and stimulated cycles to get the endometrium ready for transferring frozen embryos [8]. During this investigation, we assessed GnRH's efficiency in supporting the luteal phase in each of these four FET regimens. Patients were divided into two groups: one that received GnRH (triptorelin) during the initial stage of the luteal phase, and the other was the control group. During the luteal phase support (LPS) stage in the study group, we employed GnRH in addition to other conventional therapies. On the other hand, we didn't administer GnRH and instead employed standard LPS methods in the control group.

We assessed ovulation in participants with NCs based on each of their menstrual cycles. Transvaginal ultrasounds were performed on participants between the ninth and 10th days of their menstrual cycle. Transvaginal ultrasonography, serum estradiol (E2), and serum LH were used to track follicular growth. Transvaginal ultrasound examinations were done daily until ovulation, when the LH level was greater than 20 IU/L [9]. hCG in the amount of 5000 international units (IU) was given to initiate oocyte ovulation when the dominating follicle's average diameter was larger than 17 millimeters and LH was less than 20 IU/L. An embryo transfer was performed on the third day, during the cleavage phase, which starts on the second day of the menstrual cycle and lasts 14 days after ovulation [10].

On days 2 or 3 of the menstrual cycle, oral estradiol valerate (Progynova) at a dose of 6-8 mg was given daily for the HRT-FET cycle [11]. Transvaginal ultrasonography and serum progesterone levels were assessed after 10-12 days. When the thickness of the endometrium was at least 7 millimeters, a progesterone dose of 200 mg was given vaginally three times a day. When the serum progesterone was 1.5 ng/mL, 20 mg of dydrogesterone was administered orally twice daily for two to five days. The duration of this protocol was 14 days [12-14]. The patients were given 3.75 mg of GnRH as part of the GnRH-HRT regimen in the early days (2/3 d) of menstruation for the early follicular phase.

Regardless of their treatment condition, after 28 days, we required them to go back to the hospital [15]. The patients' ultrasound results and hormone levels were then used to determine if the patient had reached a state of pituitary downregulation. When levels of estrogen (E2) reached 183.5 pmol/L, follicle-stimulating hormone (FSH) reached 5 U/L, LH reached 5 U/L, endometrial thickness reached 5 mm, and no significant follicle or cyst was seen, the standard criteria for defining down-regulation status were applied. GnRH-HRT regimene contued for 30 days [16].

Drugs like clomiphene citrate and letrozole, with or without human menopausal gonadotropin (HMG), were used in the stimulated cycle protocol of FET to stimulate ovulation. The dose was 300~450 IU/daily and it lasted for 10 days. A 2-6 mg/day dose of endogenous estrogen and 30-35 nmol/L dose of progesterone helped to get the endometrium ready [17].

Embryo Thawing Transfer

We defrosted D3 embryos using customary methods for vitrification, and transplanting was done when more than 50% of the blastomeres survived following thawing.

Luteal Support Method: Frozen-Thawed Embryo Transfer

We began providing dydrogesterone on the second day following ovulation, depending on the needs of each patient. Some patients preferred oral drugs, some requested injections, and yet others used vaginal suppositories. Up until 14 days following transplantation, the dosage was as follows: 20 mg/d orally and 60-80 mg/day of progesterone by injection, or 200 mg twice daily via vaginal suppository.

For the GnRH group, triptorelin acetate (France), 0.1 mg/dose, was injected subcutaneously once on the fourth or sixth day four times following oocyte retrieval (after ovulation) in the basic addition of progesterone and dydrogesterone. After that, triptorelin was terminated, while other LPS treatments were continued until the 12th week of pregnancy. In the control group, only existing luteal support medications without the addition of triptorelin were continued to be taken during the same period of pregnancy.

On days 35, 55, and 75, the second, third, and fourth pregnancy tests were performed. All luteal support drugs ceased being administered once an ectopic pregnancy was determined to be present or when the pregnancy wasn't found during the test.

Observation Indicators and Follow-up

After 14, 35, 55, or 75 days following transplantation, patients successfully completed an HCG blood serum pregnancy test. They were followed up till the delivery. We looked at their LBRs and CPRs.

Statistical analysis

For statistical analysis, we used IBM SPSS Statistics for Windows, Version 26.0 (Released 2019; IBM Corp., Armonk, New York, United States). Continuous data were reported as means ± SD. To establish the statistical significance of percentages and ORs, we compared the averages using cross-tabs, performed a Chi-square test, and calculated risk estimates. We defined the significance of statistics as P < 0.05 and an OR greater than 1.

## Results

We looked at a total of 3518 cycles, 1033 of which were in the study group and were given triptorelin three to four times in the basic LPS following embryo transfer. Of these 1033 cycles, 587 were noted for CPR and 531 for LBR. The control group had a total of 2483 cycles; 1277 of those cycles had clinical pregnancies, while 1129 of those cycles had LBRs reported. They were all treated using the standard practice of LPS after embryo transfers.

Table 1 lists the basic patient characteristics. Age, BMI, duration of infertility, AMH, and antral follicle count (AFC) had no significant difference in the two groups.

**Table 1:** Contrast of basic indicators between two groups AMH: Anti-Mullerian hormone; AFC: Antral follicle counts; GnRH: Gonadotropin-releasing hormone

Items	GnRH Group (n=1033)	Non-GnRH Group (n=2485)	P-value
Age (years)	33.32±5.62	33.46±5.56	0.518
BMI (kg/m^2^)	23.64±3.59	23.68±23.68	0.76
Duration of infertility (years)	4.49±3.46	4.47±3.51	0.864
AMH (ng/ml)	4.36±4.34	4.36±4.27	0.955
AFC	20.61±12.32	21.56±44.53	0.468

As shown in Table 2, there were no significant differences between the two groups in terms of endometrial thickness. However, the total number of transferred embryos was found to be lower in the GnRH group than in the non-GnRH group.

**Table 2:** Comparison of transfer of embryos in two groupings GnRH: Gonadotropin-releasing hormone

Items	GnRH Goup (N=1033)	Non-GnRH Group (N=2485)	P-value
Endometrial thickness (mm)	9.84±1.92	9.8±2.03	0.598
Total number of transferred embryos	1.73±.444	1.78±0.414	0.001

Table 3 presents the outcomes after embryo transfer. For HRT-FET cycles, we discovered important differences in frequencies for clinical pregnancy (58.00% versus 48.40%, P-value = 0.003) and live births (52.7% vs. 45.6%, P = 0.003) between the two groups. CPR for the NC-FET, GnRH+HRT-FET, and stimulation-FET cycles had no significant difference in the above two groups (58.20% versus 52.94%, P-value =0.364, 53.00% versus 53.00%, P-value =0.176, and 59.30% versus 52.90%, P-value =0.566, respectively). LBR for these two groups had no important differences for NC-FET, G.n.R.H-a+HRT-FET, or stimulation-FET cycles (54.40% versus 47.00, P-value=0.211, 46.20% versus 47.30%, and 59.30% versus 47.10%, P-value=0.247, respectively). In the NC-FET group, the OR for clinical pregnancy following HRT-FET cycles was 1.47, 95%CI 1.24-1.75, and it was highly significant (P-value =0.003). CPR increased by 47% in the GnRH-HRT group. In the same group, the OR for live birth during HRT-FET cycles was 1.33, 95%CI 1.12-1.57, and it was significant (P-value =0.001). LBR increased by 33% in the GnRH group.

**Table 3:** Comparison of pregnancy outcomes between the two groups FET: Frozen embryo transfer; CPR: Clinical pregnancy rate; LBR: Live birth rate; NC: Natural cycle P-value<0.05: significant difference

Items	GnRH group (n=1033)	Non-GnRH group (n=2485)	P-value	OR	95% CI	Increment/Decrement
CPR (all FET)	56.8% (n= 587)	51.4% (n=1277)	0.003	1.24	1.08-1.44	24%
LBR (all FET)	51.4% ( n=531)	45.4% ( n=1129)	0.001	1.27	1.10-1.47	27%
NC-FET						
CPR	58.2% (n=46)	52.9% (n=1818)	0.364	1.24	0.79-1.95	24%
LBR	54.4% (n=43)	47.0% (n=1617)	0.211	1.35	0.86-2.11	35%
HRT-FET						
CPR	58.0% (n=391)	48.4% (n=1338)	0.003	1.47	1.24-1.75	47%
LBR	52.7% (n=355)	45.6% (n=1262)	0.001	1.33	1.12-1.57	33%
GnRH+HRT-FET						
CPR	53.0% (n=134)	53.0% (n=1730)	0.176	1	0.77-1.29	0.0%
LBR	46.2% (n=117)	47.3% (n=1543)	0.794	0.96	0.74-1.24	-4%
Stimulation FET						
CPR	59.3% (n=16)	52.9% (n=1848)	0.566	1.30	0.6-2.79	30%
LBR	59.3% (n=16)	47.1% (n=1644)	0.247	1.64	0.76-3.53	64%

## Discussion

In four of the FET cycles, HRT-FET appears to be the most effective cycle protocol in terms of CPR and LBR when triptorelin doses are administered during the luteal phase compared to the old luteal phase treatment [18].

The embryo's quality and the endometrium’s receptivity are key parameters that affect the success rate of a frozen-thawed embryo transfer [14]. NCs, HRT cycles, GnRH+HRT cycles, and stimulation cycles can all be used to prepare the endometrium [14,19,20].

For embryo implantation and pregnancy maintenance, the corpus luteum must function normally. Controlled ovarian stimulation-related corpus luteum dysfunction can result in a low pregnancy rate, a low embryo implantation rate, and a high rate of early miscarriage [21]. As a result, clinical research on the luteal support drugs used in ART treatment is becoming quite popular. Although LH secretion in the luteal phase can partially rebound after GnRH is stopped, progesterone synthesis may not be raised. Endometrial biopsy evidence shows that once the endometrial development sheds off, the development of glandular cells slows down following the administration of GnRH in the middle of the luteal phase. Progesterone levels falling will have an impact on both uterine contraction and endometrial growth. A high frequency of uterine contraction during transplantation can impair embryo placement, prevent implantation, and lower pregnancy rates, according to research using ultrasound to assess the frequency and direction of uterine contraction [22].

Some researchers reported administering a 0.1 mg dose of GnRH agonist as luteal support during the sixth day directly after fertilization [23,24]. These results showed that this treatment significantly enhanced clinical outcomes like implantation rates, pregnancy rates, and birth rates when compared to placebo. This improvement may be explained by the combined effects of GnRH on the embryos and corpus luteum [23]. Some researchers employed GnRH successfully as luteal support for in vitro fertilization and embryo transfer (IVF-ET) treatments and intrauterine artificial insemination, and they hypothesized that GnRH would also be useful in ART [23,25]. GnRH can boost other pregnancy-related peptides released by the corpus luteum, like relaxin, in addition to just raising progesterone and E2 levels in the blood. LH may directly affect the endometrium, causing it to release cytokines and angiogenic substances that are helpful for embryo implantation. Additionally, it may directly act on the embryo and encourage its development because trophoblastic cells contain GnRH receptors [26].

The endogenous corpus luteum is at its lowest stage six days following egg retrieval. At this point, GnRH is used as the corpus luteum's primary support. It binds to the pituitary gland's newly produced GnRH receptor, generating a "flare-up" effect that increases the secretion of the ovarian hormones FSH and LH. Increased LH causes granulocytes to secrete more progesterone, which improves ovarian luteal function and makes pregnancy more likely to develop and remain so [27].

Early investigations revealed GnRH receptor expression in maternal endometrium and human embryonic trophoblast cells. According to one study, functional LH receptors had been identified in human uterine tissue, which raises the possibility that using GnRH during the mid-luteal phase will enhance the likelihood of clinical pregnancy and facilitate embryo implantation [28]. Razieh et al. reported that a single injection of GnRH in the luteum phase increased CPR and embryo implantation compared to the standard luteal support group [29].

Human embryos and endometrial stromal cells both have GnRH receptor mRNA, and giving GnRH during the mid-luteal phase may encourage early implantation embryos to secrete hCG. Studies from recent years have suggested using GnRH as luteal support; however, the sample size is relatively small. Future discussions will focus on how the LPS differs from the fresh cycle and how the success rate in freeze-to-thaw embryo transfer cycles has enhanced because of advances in freeze-thaw technology [30].

Patients who underwent all four FET cycles were chosen for investigation. CPR and LBR of the GnRH (triptorelin) group were 47% and 33% greater than those of the group without GnRH addition, respectively, and had significant differences statistically based on the results of HRT-FET cycles. There are ongoing studies on whether giving GnRH in the luteal phase increases the chance of abnormal fetal births.

In this study, additional monitoring of the mothers and fetuses was done to see if the use of GnRH during the luteal phase raised the risk of fetal birth abnormalities.

Limitations

As it is a retrospective cohort study, possible biases cannot be ruled out and some key statistics may be missed; therefore, it is recommended to do a large-scale trial for the verification of our findings.

## Conclusions

CPR and LBR can rise when GnRH is added during luteal phase of HRT-FET, and it may also open up new possibilities for luteal support. This study is, however, a small-scale one. To further compare the variations in the use of GnRH in various freeze-thaw schemes, the selection of treatment population, the dose of GnRH used, the time and frequency of administration, and to obtain a unified standard for the effective luteal support of GnRH, it is suggested that a randomized control trial be done on a large sample. At the same time, we must also consider how GnRH use affects fetuses.
